# A Comparative Finite Element Analysis of Titanium, Autogenous Bone, and Polyetheretherketone (PEEK)-Based Solutions for Mandibular Reconstruction

**DOI:** 10.3390/ma18020314

**Published:** 2025-01-12

**Authors:** Ionut Gabriel Ghionea, Cristian Ioan Tarba, Corina Marilena Cristache, Iulian Filipov, Irina Adriana Beuran

**Affiliations:** 1 Manufacturing Engineering Department, Faculty of Industrial Engineering and Robotics, National University of Science and Technology Politehnica Bucharest, 313 Splaiul Independentei, 060042 Bucharest, Romania; gabriel.ghionea@upb.ro (I.G.G.); cristian.tarba@upb.ro (C.I.T.); 2 Department of Dental Techniques, “Carol Davila” University of Medicine and Pharmacy, 8, Eroii Sanitari Blvd., 050474 Bucharest, Romania; irina.beuran@umfcd.ro; 3 Doctoral School, “Carol Davila” University of Medicine and Pharmacy, 37 Dionisie Lupu Street, 020021 Bucharest, Romania; iulian.filipov@drd.umfcd.ro

**Keywords:** mandibular reconstruction, finite element analysis, titanium customized plates, PEEK customized plates, autogenous bone

## Abstract

Mandibular reconstruction is essential for restoring both function and aesthetics after segmental resection due to tumoral pathology. This study aimed to conduct a comparative analysis of three reconstruction strategies for defects resulting from segmental mandibular resection, utilizing finite element analysis (FEA). Methods: A digital model of the mandible was created from CBCT data and optimized for FEA. Three reconstruction scenarios were simulated: fixation with a titanium plate, reconstruction with an autogenous fibular graft stabilized with the same titanium plate, and fixation with a customized PEEK plate. Various plate thicknesses were analyzed to determine the stress and deformation patterns under masticatory loads. Results: Titanium plates provided superior mechanical stability but showed stress concentrations near screw fixation points. The addition of autogenous bone grafts reduced stress on the plate and improved structural integrity. PEEK plates exhibited reduced stress shielding and better load distribution, but thinner designs were prone to deformation. Minimum recommended thicknesses of 1.2 mm for titanium plates and 1.8 mm for PEEK plates were identified by FEA. Conclusions: This study highlights the importance of material selection and patient-specific design in mandibular reconstruction. Autogenous bone grafts combined with titanium plates demonstrated the best biomechanical outcomes, while PEEK plates offer a promising alternative, particularly for patients where grafting is contraindicated.

## 1. Introduction

Oral cancer, which accounts for approximately 377,700 new cases yearly worldwide, as reported for 2020 [1], frequently involves tumors in the mandible, necessitating extensive surgical resection of affected bone and surrounding soft tissue to ensure negative margins. This form of head and neck cancer not only compromises the patient’s aesthetic appearance and comfort, but also severely impacts essential functions, such as mastication, speech, and breathing [2]. Besides the malignant tumors, ameloblastoma, a benign odontogenic tumor of epithelial origin, yet locally aggressive, primarily arising in the mandible, often requires segmental surgical resection due to its propensity for recurrence [3]. 

Segmental mandibular resections without reconstruction may cause the mandible to deviate toward the resected side, resulting in severe functional impairments and significant cosmetic deficiencies [4].

Mandibular defects resulting from tumor excisions present unique challenges in reconstructive surgery, as the mandible is a movable, load-bearing bone essential for facial structure, aspect and function. Traditional reconstruction approaches often rely on autologous tissue, such as fibula or iliac crest grafts, which provide structural stability and allow for prosthetic reconstruction. Free fibula osteo-cutaneous flap is considered the treatment of choice for mandibular reconstruction [5]. However, this approach may not be feasible in certain situations, such as when there are extensive soft tissue defects necessitating large osteotomies, presence of peripheral vascular disease affecting the lower limbs, venous insufficiency, history of deep vein thrombosis, prior lower limb amputations, or patient refusal of the procedure [6]. Additionally, mandibular reconstruction often requires collaboration among multiple surgical teams, including orthopedic and plastic surgeons, to perform complex microvascular anastomoses essential for the success of the free flap or for graft harvesting [7]. Standard bone graft fixation often employs commercially available titanium plates and screws of standard or customized sizes and designs. Moreover, due to individual variations in patient anatomy and bone structure, these stock plates may not align precisely with the resected mandibular region, and the grafted bone fragments, leading to challenges in achieving optimal surgical outcomes [8]. In certain cases, titanium plates are employed to unite the remaining mandibular fragments following resection. This approach is particularly considered when autogenous bone grafts are not feasible [9]. However, relying solely on titanium plates without bone grafts can lead to complications, including plate fracture, screw loosening, and bone resorption, which may compromise the long-term success of the reconstruction [10].

To address the limitations of standard mandibular reconstruction plates, patient-specific implants can be designed using data from computer tomography (CT) or cone beam computer tomography (CBCT) scans. Employing computer-aided design/manufacturing (CAD/CAM) technology allows for the design and creation of customized plates that conform precisely to an individual’s anatomy, enhancing surgical outcomes. 

Titanium and its alloys are commonly used materials for both standard and customized mandibular plates due to their strength and biocompatibility. However, titanium implants can cause significant streak and blooming artifacts in CT images, hindering the accurate radiological assessment of surrounding tissues [11]. This issue complicates postoperative tumor monitoring and affects the precision of adjuvant radiotherapy, which relies on high-resolution, artifact-free planning CT scans [12]. Additionally, titanium implants can lead to radiation-dose enhancement in adjacent tissues during radiotherapy [13]. 

As an alternative, polyetheretherketone (PEEK) has emerged as a promising material for mandibular reconstruction plates. PEEK offers favorable biocompatibility and possesses a Young’s modulus of approximately 3.9 GPa, closely matching that of cortical bone [14,15]. This similarity in mechanical properties reduces stress shielding and promotes better loads distribution, potentially improving the longevity and functionality of the implant. Moreover, PEEK’s radiolucency minimizes imaging artifacts, facilitating more accurate postoperative assessments and treatment planning [12].

Finite element analysis (FEA) has been extensively utilized to simulate mandibular movements and assess reconstruction strategies following resection [2]. However, significant gaps remain in current research, particularly concerning the comparative performance of titanium and PEEK materials in customized shape plates and their effects on stress distribution and structural integrity. Moreover, studies investigating the potential replacement of autogenous bone grafts with solely customized plates, such as titanium or PEEK, are limited, despite the growing interest in PEEK’s biomechanical and clinical advantages [16]. Additionally, few studies adopt a comprehensive approach to mandibular reconstruction, encompassing mandible modeling, custom plate design and fabrication, FEM development, and simulations, along with illustrative video demonstrations of the entire workflow and results.

This study aimed to conduct a comparative analysis of three reconstruction strategies for defects resulting from segmental mandibular resection: a patient-specific titanium plate, autogenous bone stabilized with a titanium plate, and a patient-specific PEEK plate, utilizing finite element analysis (FEA). A secondary objective was to determine the optimal plate thickness in each case to ensure structural integrity during masticatory movements.

## 2. Materials and Methods

**Figure 1:**
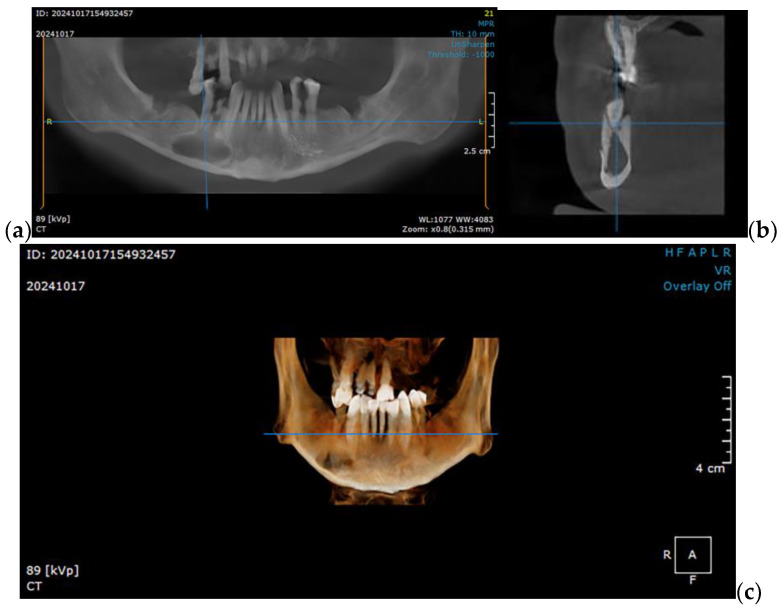
CBCT imaging showcasing a right mandibular tumor initially diagnosed as a residual cyst: (**a**) coronal view, (**b**) sagittal view, and (**c**) 3D reconstruction, illustrating the lesion’s extent and its anatomical relationships in detail.

The present study utilizes a simulation to determine the optimal option for mandibular reconstruction in clinical scenarios similar to the following case of a 49-year-old partially edentulous patient with a significant history of smoking (15–20 cigarettes daily) and chronic alcohol consumption. The patient has no documented comorbidities or prescribed medications but exhibits a markedly thin and mildly cachectic physique. Over the preceding eight months, the patient experienced three episodes of abscess formation in the right mandibular vestibule. The initial diagnosis was a radicular cyst (Figure 1), for which enucleation and extraction of all hopeless teeth was performed. Subsequent histopathological examination confirmed the presence of an ameloblastoma, which was further validated through immunohistochemical analysis. This clinical condition necessitates surgical intervention involving segmental mandibular resection, followed by reconstruction.

To explore various reconstruction options for a right segmental mandibular reconstruction following ameloblastoma ablation, this study utilized a STL (Standard Tessellation Language) file of a healthy adult male mandible. The file was generated by segmenting the mandible from CBCT scan in DICOM (Digital Imaging and Communications in Medicine) format. To ensure suitability for finite element method (FEM) simulation, the mandibular geometry was simplified to balance computational efficiency with anatomical precision. This simplification was carried out using CATIA V5R21 (Computer-Aided Three-Dimensional Interactive Applications, Dassault Systèmes, Vélizy-Villacoublay, France), providing an optimized model for accurate simulation analysis. 

### 2.1. Optimizing the Mandibular Model for Simulation

Once imported into CATIA V5R21, the STL file undergoes an initial surface validity check, followed by a simplification process using the *DMU Optimizer* workbench. This workbench offers several options applicable depending on the complexity and shape of the analyzed surface.

**Figure 2:**
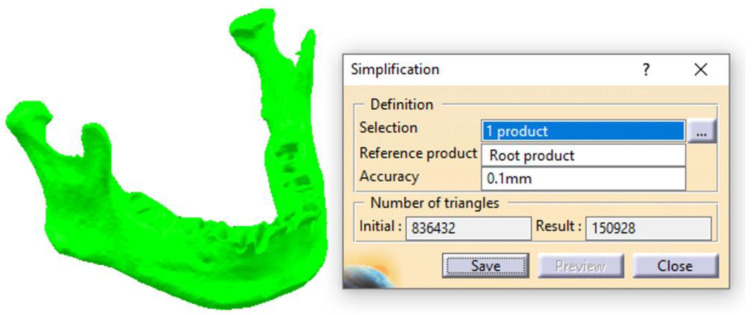
The first stage of simplifying the scanned surface of the mandible.

By using the *Simplification* tool, the scanned surface of the mandible, which initially contained 836,400 triangles, is optimized and reduced to 151,000 triangles (Figure 2). This optimization makes the 3D model much more suitable for subsequent stages and, ultimately, for FEA. In this study, an accuracy of 0.1 mm was used; however, excellent results can also be achieved with higher *Accuracy* parameter values (up to 0.4 mm). This parameter directly influences the number of triangles in the *Result* field, determining a more or less refined surface. For *Accuracy* = 0.1 mm, the surface retains a relatively high level of complexity for a model of the mandible’s dimensions, but it allows for subsequent editing and refinements necessary to create an accurate CAD model.

**Figure 3:**
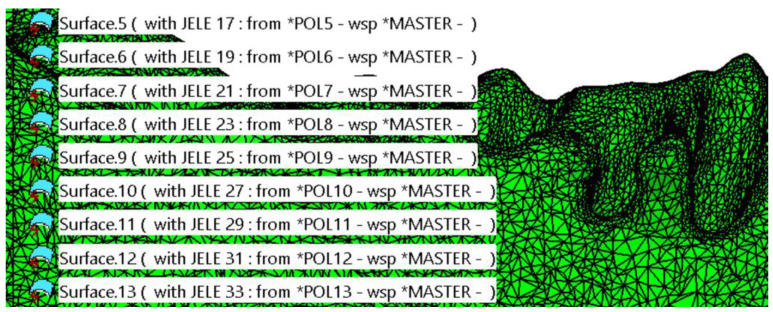
Initial simplification of the model. The surface of the mandible is covered with a high number of patches composed of triangles.

Following this initial simplification, a file in .*model* format is obtained, consisting of approximately 150 distinct patches, each containing hundreds or thousands of triangles (Figure 3). These triangles are very small in size (ranging from 0.3 to 1.5 mm along the edges) and are distributed across the entire surface of the mandible. According to the file type and the recommended workflow for complex surfaces, the transformation of these patches into solid bodies is deferred. This decision is due to the need for additional advanced editing and refinement stages.

Several steps were carried out with the surface, which initially possessed only area properties and no volumetric attributes. Before grouping all the patches (with areas ranging from approximately 10 mm^2^ to 200 mm^2^) into a single entity, careful attention was devoted to ensuring the accuracy of the surface. This process included verifying that the patches were connected, free from self-intersections, without missing triangles, and devoid of errors resulting from file export–import operations, among other potential issues.

**Figure 4:**
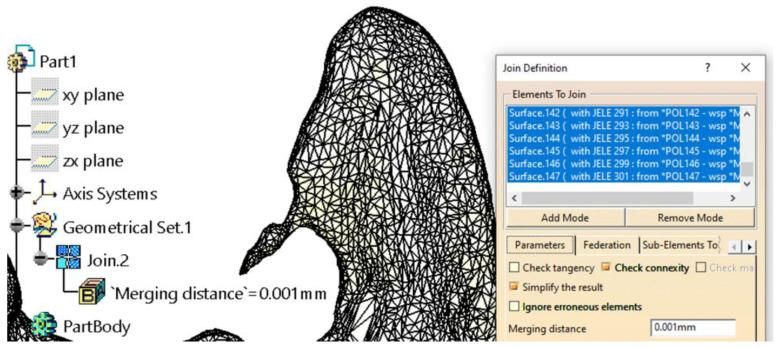
Further model simplification Grouping the patches into a single surface. An example of a scan error was found in the coronoid apophysis area.

Grouping the patches into a single surface (using the *Join* tool in the *Generative Shape Design* workbench) is necessary for several reasons, two of which are particularly important: it significantly simplifies and secures manipulation while providing cohesion to the 3D model. Additionally, for the model to be transformed into a solid body, it must present as a single, closed surface that is geometrically correct. Depending on the computational power of the system used for the whole workflow, the grouping process may take anywhere from a few minutes to over an hour, as the CATIA V5R21 software verifies each patch individually and its connections to adjacent patches. This step is followed by a secondary simplification of the resulting surface (Figure 4). This simplification does not reduce the number of triangles but instead corrects tangency conditions between triangles connected by edges, adjusting their sizes to better cover the resulting surface.

Some additional editing is necessary to address imperfections caused by scanning. For example, in the left coronoid apophysis area (Figure 4), such an issue is evident. It is well known that an anatomic surface of such complexity, when scanned and imported into a CAD software (CATIA V5R21)—regardless of the software’s manufacturer or capabilities—requires manual editing and corrections to achieve a proper model suitable for finite element analysis (FEA).

**Figure 5:**
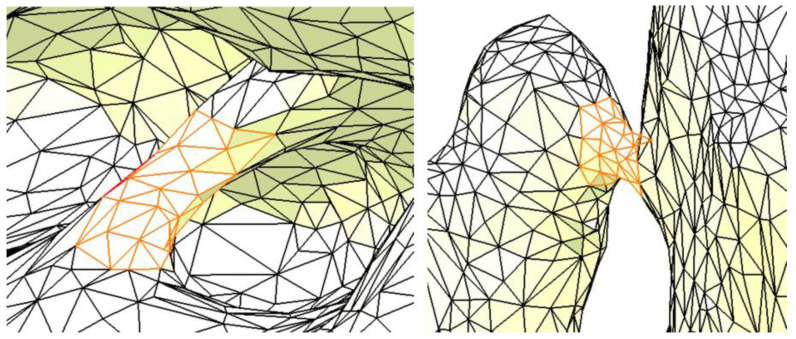
Two examples of imperfections in the resulting surface. The orange color highlights the imperfections.

Figure 5 presents two additional examples of imperfections: the one on the left is located in the area of the interradicular septum, while the one on the right is in the region of the canine–canine alveolar process. These imperfections are due to the segmentation of the mandible without considering the teeth. Although such imperfections may not significantly affect the results of the FEA, they may cause issues during the creation and discretization of the FEM model in CATIA V5R21, potentially rendering the analysis difficult or impossible. Therefore, if these imperfections are identified and can be addressed, they should be removed or repaired.

The surface obtained using the *Join* tool must be converted into a mesh entity to perform these corrections. The *Shape Sculptor* workbench provides numerous tools for editing the mesh at the point, edge, and triangle levels. Among these, the *Tessellate* tool is first used with a sag value of 0.05 mm. This sag value represents the maximum distance or deviation between the geometry and the triangles, thereby simplifying and refining the mandible surface.

**Figure 6:**
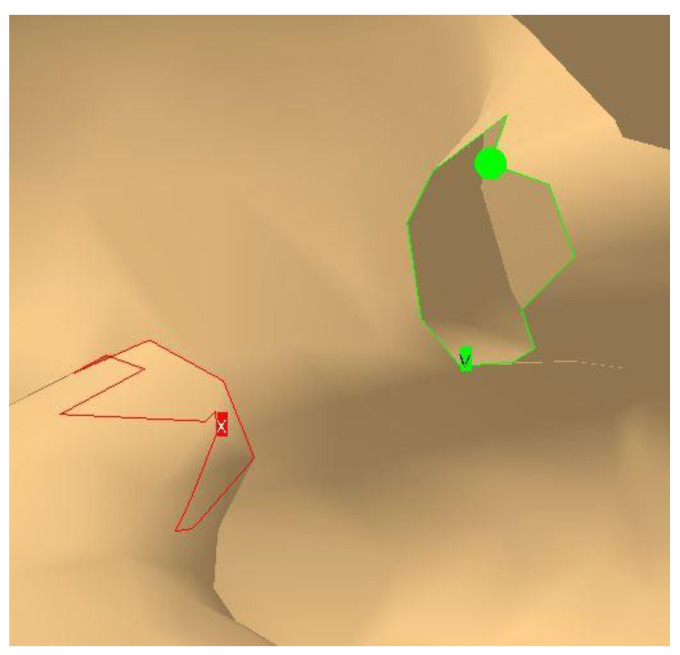
Closing a gap by deleting and refining the mesh.

The tessellation process is crucial and mandatory, as it calculates a geometric discretization of curves, surfaces, bodies, edges, and faces. Once tessellated, these entities are transformed into discretized data, enabling their use for visualization, mesh editing, reconstruction, and more. After the tessellation step, all imperfections are either edited or removed (by deleting or adding triangles, as shown in Figure 6) using the *Remove Element* and *Collapse Element* tools in the *Digitized Shape Editor* workbench.

Figure 6 illustrates two areas where numerous triangles have been removed. These resulting gaps must be identified, closed, and verified using the *Fill Holes* and *Mesh Cleaner* tools. This step is essential for the proper creation of the final mandible mesh. The *Mesh Cleaner* tool is particularly effective at detecting and repairing surface issues that are difficult or nearly impossible for the user to identify manually.

**Figure 7:**
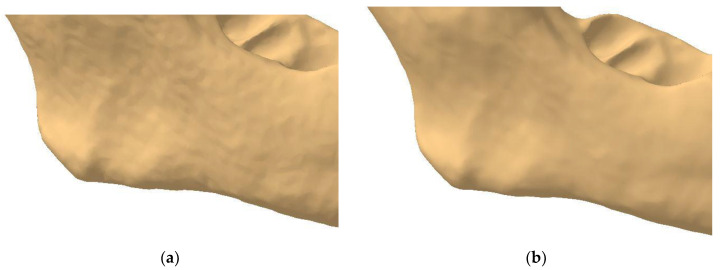
The rough (**a**) and smooth (**b**) surfaces of the mandible.

Being automatically generated through scanning and without surface-level editing, the mandible initially appears as shown in Figure 7a. The surface is rough, with an excessive number of triangles. If the surface were to remain in this state, the finite element analysis would include numerous unnecessary elements that do not improve the results but instead use important hardware resources and increase processing time. Therefore, it is necessary to apply the *Mesh Smoothing* tool, which smooths the surface and provides a much closer approximation to the appearance of a real mandible (Figure 7b).

Depending on the settings applied during this smoothing operation, the user also has the option to manually level the mandible’s surface using the *Brush Smooth* tool. However, care must be taken when using this tool, as it can affect the shape and details of the mandible. Its application is nonetheless essential, as certain scanning defects are not corrected by the previously used tools.

For the mandible analyzed, an excessive level of anatomical detail was observed along the alveolar ridge. Based on the authors’ experience, certain elements, such as sharp edges, were identified as requiring manual rounding to achieve better discretization of the model. This procedure is time-consuming and demands significant experience and precision to avoid damaging the resulting surface and its important details. Specific areas must be avoided with this tool to preserve their integrity (e.g., condyles, coronoid apophysis, and the mental foramen). By maintaining the complexity and specific shape of this bone, excellent results can be achieved in the subsequent FEA.

**Figure 8:**
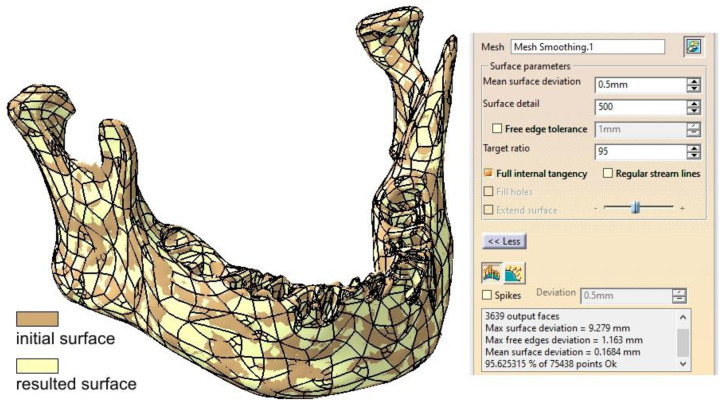
Comparison between the initial surface and the final surface obtained using the *Automatic Surface* tool.

Once the mandibular surface is finalized through these edits and corrections, it can be converted into the final surface required for solid modeling and FEM analysis. Using the *Quick Surface Reconstruction* workbench, the *Automatic Surface* tool is applied with parameters set to a mean surface deviation of 0.5 mm, surface detail of 12,000, and target ratio of 95% (Figure 8). This tool is versatile and suitable for a variety of surfaces, capable of preserving and creating complex shapes using a minimal set of Non-Uniform Rational B-Splines (NURBS). Typically, the resulting surfaces are generated with or without full internal tangency between faces.

There are certain capabilities and characteristics that recommend the use of the *Automatic Surface* tool. However, it struggles to handle very sharp edges effectively, and the input should be a mesh rather than a cloud of points. The parameters used ensure a high level of detail in the mandibular surface, along with a slight simplification achieved through the *Target Ratio* parameter. From our experience, a ratio close to 100 is overly time- and resource-consuming without providing better discretization for the proposed model.

**Figure 9:**
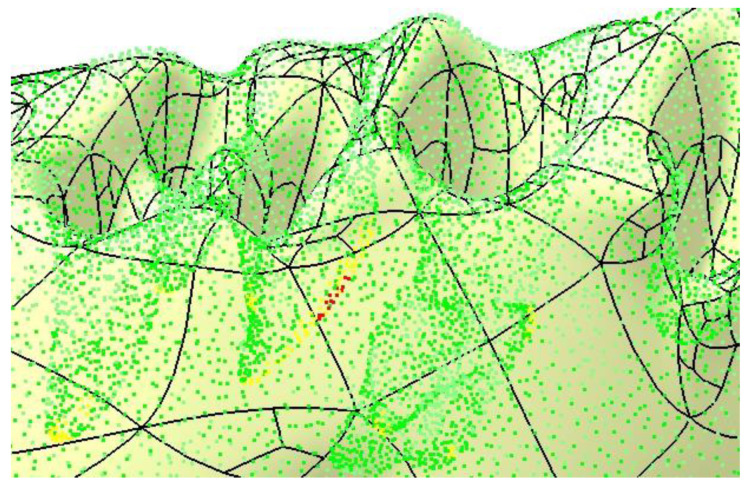
Final check of the mandibular surface.

A particularly useful option is *Full Internal Tangency*, which ensures proper tangency between the faces of the resulting surface. Figure 8 provides a comparative overlay of the initial and final surfaces, demonstrating the differences. The accuracy of the resulting surface is further confirmed by applying the *Deviation Analysis* tool, as shown in Figure 9. The yellow and red dots mark the critical spots where the authors applied corrections, some nodes of the mesh were removed (red) while some others were repositioned. These are only minor changes, but important for the integrity of the final surface.

The CATIA V5R21 software provides a range of tools and options designed for the editing, simplification, and verification of scanned surfaces. The inherent complexity of these surfaces often introduces challenges related to continuity, tangency, and coherence. Processing a scanned surface is a meticulous task that goes beyond the application of automated tools and requires detailed manual editing at the levels of points, edges, and triangles. This process also involves identifying and verifying specific areas and making precise simplifications to maintain the integrity and detail of the final result.

The correct application of this methodology allows for the generation of a solid model (using the *Close Surface* tool from the *Part Design* workbench) suitable for finite element analysis. To support this workflow, the authors have created a video that demonstrates the complete process of transforming a scanned surface into a solid model in the Supplementary Materials: https://youtu.be/DAt7YAio2A0. (accessed on 11 January 2025)

### 2.2. Post Tumor Ablation Defect Simulation

Segmental mandibular resection is often deemed necessary in the management of ameloblastoma to achieve complete tumor removal and minimize recurrence [17,18]. Immediate reconstruction post-resection has been shown to be safe and predictable, facilitating the restoration of mandibular continuity and function [19].

For defect simulation a segment of the right hemimandibular body is resected from the alveolar ridge to the basal border over a length of 22 mm, with the two proximal surfaces (mesial and distal) considered parallel.

### 2.3. Reconstruction Simulation

This study simulates three reconstruction scenarios for the mandibular defect:

#### 2.3.1. Fixation with a Titanium Osteosynthesis Plate

The remaining mandibular fragments are stabilized using a Ti-Grade-4 (TI75A) titanium alloy plate with the following physical properties: Young’s modulus of 104,000 MPa, Poisson’s ratio of 0.34, density of 4500 kg/m^3^, and yield strength of 550 MPa [20,21]. The fixing plate has a standard width of 2.5 mm, six Ø2 mm holes, and a length of 55 mm. The plate thickness can vary between 0.6 mm and 2 mm, as recommended by several studies [20,22]. Finite element analysis will evaluate these variations.

#### 2.3.2. Reconstruction with a Fibular Autograft

In the second scenario, the defect is reconstructed using an autogenous fibular graft fixed with an osteosynthesis plate to enhance mandibular rigidity.

#### 2.3.3. Fixation with a Customized Polyetheretherketone (PEEK) Plate

In the third scenario, the defect is reconstructed using a customized plate made of PEEK. The PEEK plate has the following physical properties: Young’s modulus of 3900 MPa, Poisson’s ratio of 0.42, density of 1300 kg/m^3^, and yield strength of 100 MPa [14,16]. The plate is 8 mm wide, is approximately 46 mm long, has rounded edges, and features a cutout to reduce mass and facilitate better fixation of soft tissues.

### 2.4. Testing Assembly

In this study, two plates are designed in CATIA v5 using the *Generative Shape Design* and *Part Design* workbenches, ensuring their shapes conform to the mandibular anatomy in the target region. For the titanium alloy plate, shaping it to the desired form can be achieved through plastic deformation; however, this introduces undesirable residual stresses and fracture risk [8]. Or it can be shaped by 3D printing [21,23,24] or sintering the designed customized shape, allowing the plate to precisely match the patient’s mandibular contour. The PEEK plate can be also manufactured through additive manufacturing techniques, offering significantly lower production costs compared to the titanium plate [14].

Despite the complex organic shape of the mandible, CATIA V5R21 provides the capability to generate successive design steps (profile drawings and projections), enabling the plates to achieve complete contact with the mandibular surface. This contact ensures accurate finite element analysis results and a custom fit for each patient, enhancing stability and comfort. The plates are affixed to the mandible and, when applicable, to the additional bone using Ø2 mm screws with a length of 5 mm, fabricated from the same Ti-Grade-4 (TI75A) alloy.

The modeling process for plates and screws is detailed, with dimensions and shapes determined based on prefabricated plate standards, the available literature [22], and iterative trials to eliminate design flaws. These include avoiding plates with insufficient thickness in the screw assembly area or sharp edges that could harm the patient’s surrounding soft tissue.

Additionally, the articular disc, positioned on the mandibular condyle as part of the temporomandibular joint (TMJ), is modeled with a thickness of 1 mm and assigned the following material properties: Young’s modulus of 30 MPa, Poisson’s ratio of 0.29, density of 1300 kg/m^3^, and yield strength of 5 MPa [25]. This modeling imparts elasticity and relative motion to the mandibular joints, which are essential for FEA.

The mandibular bone, the main component of the studied assembly, is modeled as compact and uniform to simplify the analysis [26,27]. Although this represents a limitation, the authors believe it does not significantly affect the results. Based on published studies [28], the bone material is assigned average physical properties: Young’s modulus of 13,000 MPa, Poisson’s ratio of 0.30, density of 1500 kg/m^3^, and yield strength of 140 MPa.

Dimensional and geometric constraints for correct component positioning are applied in the *Assembly Design* workbench. Constraints such as coincidence, surface contact, and specific distances ensure proper alignment of components like the mandible, TMJ (condyle–disc construct), fixing plate, and screws. Correct assembly positioning is critical for creating a valid finite element model, as constraints reflect the degrees of freedom for each component.

The FEA begins in the *Generative Structural Analysis* workbench. Each component is discretized into finite elements, balancing the level of detail with computational resources. From the authors’ experience, a higher level of mesh discretization typically offers accurate simulation results, although excessive element counts rarely improve accuracy [29].

Meshing involves approximating the real structure with numerous simple geometric shapes (elements), interconnected by nodes. The mesh serves as an idealized mathematical model of the physical structure. Each assembly component is discretized according to its size and shape relative to others, with key characteristics detailed in Table 1.

**Figure 10:**
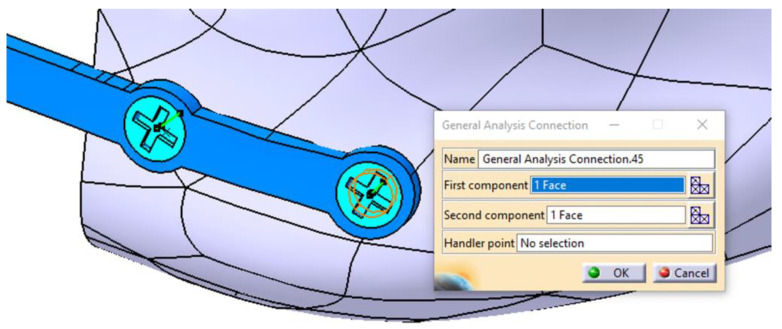
Defining the connection between the mandible, the fixing plate, and the screw.

The components of the mandibular assembly are then integrated into the FEM model by defining physical connections (Figure 10) between contacting surfaces. Given the high complexity of this model, the connections are carefully and meticulously defined. In the FEM analysis presented in this study, several types of connections were used: *Smooth*, *Rigid*, *Contact*, *General*, and *Fastened*. When implemented correctly, these connections accurately replicate real-world interactions, ensuring the reliability of the simulation.

**Figure 11:**
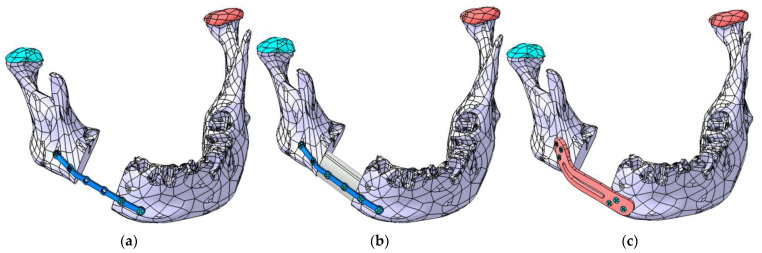
The three mandibular reconstruction options analyzed using FEA: (**a**) mandibular fragments stabilized with a Ti-Grade-4 (TI75A) plate, (**b**) autogenous fibula graft secured with a Ti-Grade-4 (TI75A) plate, and (**c**) mandibular fragments stabilized with a PEEK plate.

The primary objective of the FEA simulation is to determine, through successive iterations, the optimal shape, position, and thickness of each plate. Figure 11a–c illustrate the three cases analyzed using FEA.

The upper surface of the articular disc is constrained such that the nodes located on it do not allow any displacements or rotations, thereby restraining all six degrees of freedom. Multiple forces are applied to the mandibular surface to simulate the actions of the masseter, temporalis, and pterygoid muscles, as well as the suprahyoid musculature. To simplify the analysis, an edentulous mandible was considered. In this case, after restoring continuity, the mandible is assumed to be rehabilitated with a removable prosthesis.

**Figure 12:**
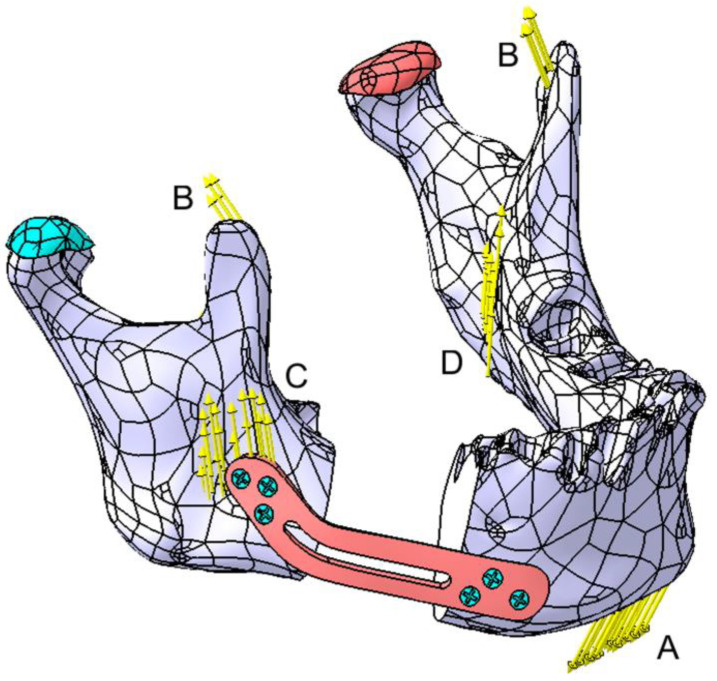
Graphical representation of the forces applied to the mandible. Zones A to D represent the forces applied by the masticatory muscles as follows: A—the suprahyoid muscle group, B—the temporalis muscle, C—the masseter muscle, and D—the medial pterygoid muscle. The articular disk is marked in green on the affected side and in red on the contralateral side.

Four zones (A–D) were identified for forces application on the mandible, as shown in Figure 12. Forces at points B, C, and D are considered symmetrical. Force A, representing the suprahyoid muscle group, is applied to the mental region, with a value of 30 N. In Zone B (temporalis muscle), two forces of 12 N each are applied; in Zone C (masseter muscle), two forces of 40 N each are applied; and in Zone D (medial pterygoid muscle), two forces of 30 N each are applied. The direction of the forces closely follows the direction of the muscular insertions; however, these forces can be resolved into the X, Y, and Z components of a Cartesian coordinate system.

The FEM analysis was performed on a computer with a 13th Gen Intel Core i7-13620H processor (2.40 GHz), a 64-bit Windows 10 operating system, x64-based architecture, and 16 GB of RAM.

## 3. Results

The initial results of the FEA focus on the most unfavorable scenario, where the two parts of the mandible are fixed solely using a Ti-Grade-4 (TI75A) plate, as shown in Figure 11a. According to the previously published studies [22], the authors suggest an average plate thickness of 2–2.5 mm. Considering that this parameter affects both the aesthetic appearance of the mandible and the patient’s comfort, successive FEM iterations were used to determine the stresses and displacements induced in all components of the assembly.

### 3.1. Fixation with a Titanium Osteosynthesis Plate

This study starts with an initial plate thickness of 2 mm, reducing it by 0.2 mm in each iteration. The maximum stress and displacement values are presented in Table 2, which also includes the maximum stresses identified in other components. The table highlights significant displacement values for both the mandible and the fixing plate, with the plate moving in unison with the mandible and the maximum displacement occurring in the chin region.

**Figure 13:**
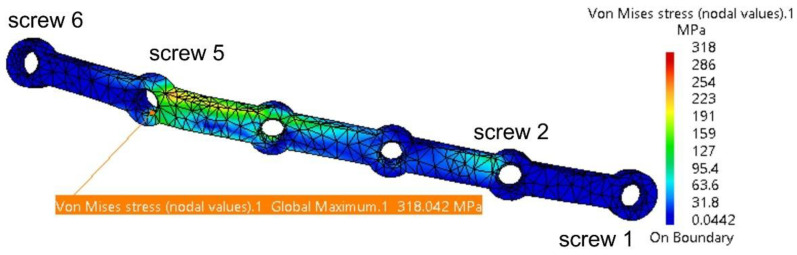
Stress distribution on the fixing plate in the assembly area with the four screws and the mandible.

Under applied forces, the fixing plate deforms and experiences torsion, with the highest stress concentrated around Screw 5, as shown in Figure 13. The risk of plastic deformation and fracture begins at a plate thickness of 1.2 mm. The maximum stress of 431 MPa is below the titanium alloy’s yield strength of 550 MPa. However, considering masticatory forces, potential computation errors for the plate (11.8%), and material fatigue, the authors recommend a minimum plate thickness of 1.2 mm.

CATIA V5R21 also identifies certain computation errors, with the 11.8% margin deemed acceptable, representing the difference between the ideal model used in FEM analysis and the real patient model. Additionally, the variation in stress distribution within the plate is noteworthy. These variations are accurate, as the plate’s thickness and shape change in each iteration, directly influencing stress distribution patterns. Thus, stresses are distributed differently for each iteration.

Table 2 shows that the stresses computed for the mandible, the two TMJs, and the screws are relatively low compared to the yield strengths of the materials used for these components.

**Figure 14:**
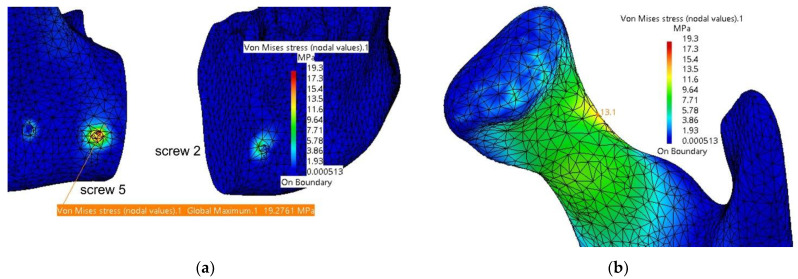
Stresses distribution on the mandible in the assembly area (**a**) with the fixing plate and screws and (**b**) in the left condyle area.

In Figure 14, the computed stresses for the mandible are shown in the assembly area with the fixing plate and screws and in the left condyle region (superior border of the condyle’s neck). The maximum values are located inside the hole corresponding to Screw 5; however, these do not pose any concerns regarding the integrity of the bone in that area. Screw 5 is also affected, but the stresses are very small compared to the yield strength of the titanium alloy.

**Figure 15:**
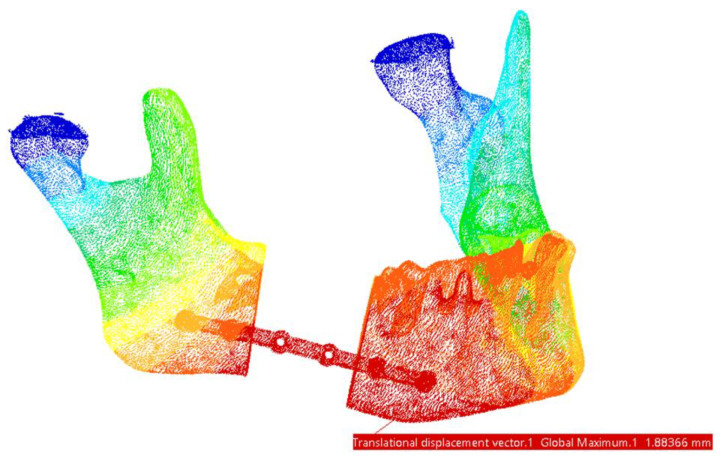
Areas with maximum displacements. The highest values are marked with red and the lowest displacements areas are marked with dark blue.

Regarding the displacements observed in this FEM model, the maximum values are located in the mandible, specifically in the chin region—at the assembly with the fixing plate and Screws 1 and 2, as shown in Figure 15. 

The use of an osteosynthesis plate alone is a viable option for mandibular reconstruction; however, incorporating an autogenous bone graft provides a superior solution, offering enhanced structural support and promoting better long-term outcomes.

### 3.2. Reconstruction with a Fibular Autograft

**Figure 16:**
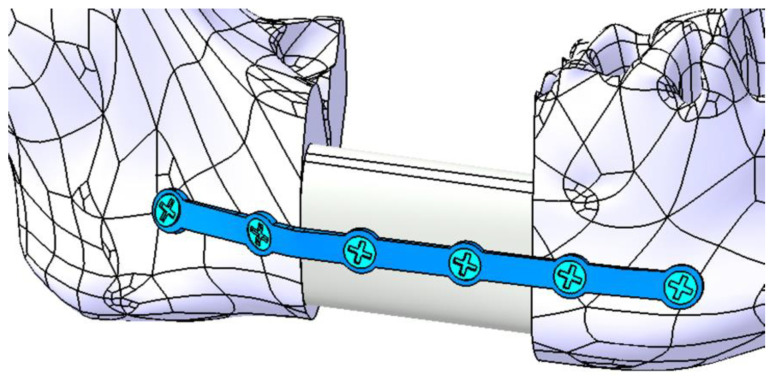
Mandibular defect reconstructed with an autogenous bone graft secured with a titanium osteosynthesis plate and six screws (four inserted in mandible and two inserted in the bone graft).

For the second simulation, the mandibular defect resulting from the resection was reconstructed using an autogenous bone graft harvested from the fibula. The graft was modeled to match the specific dimensions and shape characteristic of fibular bone. It was secured in the defect area with the same Ti-Grade-4 (TI75A) alloy plate and stabilized using two screws, as illustrated in Figure 16.

The conditions necessary for the finite element analysis simulation remain consistent with those outlined previously. However, they are supplemented by specific physical constraints applied to the grafted bone and the two additional screws. The fixing plate is assumed to have a thickness ranging between 1.2 mm and 1.8 mm, with the majority of the stresses being distributed to the grafted bone. Table 3 provides an overview of the maximum stresses and displacements observed within the assembly.

**Figure 17:**
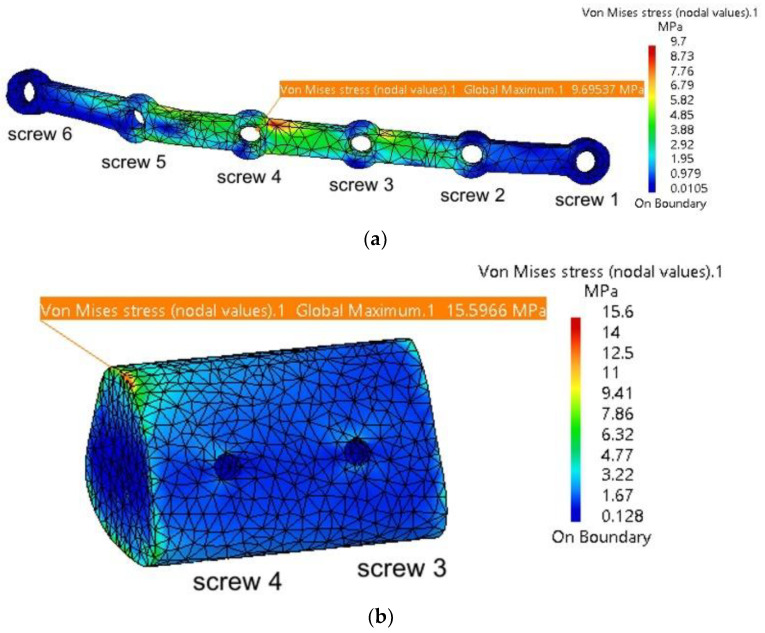
Mandibular defect reconstructed with an autogenous bone graft secured with a titanium osteosynthesis plate and screws: (**a**) stress distribution on the fixing plate in the assembly area with the six screws, mandible, and grafted bone; and (**b**) stress distribution on the grafted bone.

It is observed that the maximum stresses are significantly lower compared to those computed in the previous simulation, without the autogenous bone graft. Figure 17a shows the stress distribution within the plate model, with the highest values located near Screw 4, at the contact point with the grafted bone fragment. This fragment, illustrated in Figure 17b, exhibits maximum stresses at the interface with the mandible, and this is what the authors expected. When securely fixed and integrated into the assembly model, the autogenous bone graft absorbs stresses, preventing deformation of both the fixing plate and the mandible. Its role is particularly significant, as demonstrated by the simulation, as it prevents twisting/torquing of the plate and high displacements of the mandible, and, consequently, it reduces the stress within them.

As presented in Table 3, the simulation results show slight variations in values with each iteration, while all stresses remain well below the yield strengths of the materials used for the individual components. The maximum stresses observed in the mandible are concentrated in the same region of the left TMJ (condyle–disc construct), as noted in the previous simulation.

### 3.3. Fixation with a Customized PEEK Plate

A third simulation was also conducted, this time without using autogenous bone. Instead, the fixing plate was completely redesigned with a different shape and dimensions and was supposed to be manufactured from biocompatible PEEK material. As depicted in Figure 12, six screws were used, positioned at the extremities of the plate. These screws are arranged in a triangular configuration to better constrain all six degrees of freedom for both the plate and the mandible. Additionally, some of the stresses determined through FEM analysis are absorbed by the screws.

The discretization, material properties, and loading conditions for this new model are similar to those in the two previous simulations. The thickness of the PEEK plate varies between 1.4 and 2 mm. Table 4 presents the maximum stresses and displacements for the assembly.

The values presented in Table 4 indicate that the PEEK plate develops stresses close to the yield strength of the material used. It is recommended to use such plates with a minimum thickness of 1.8–2 mm, as thinner plates present a higher risk of plastic deformation and/or fracture.

**Figure 18:**
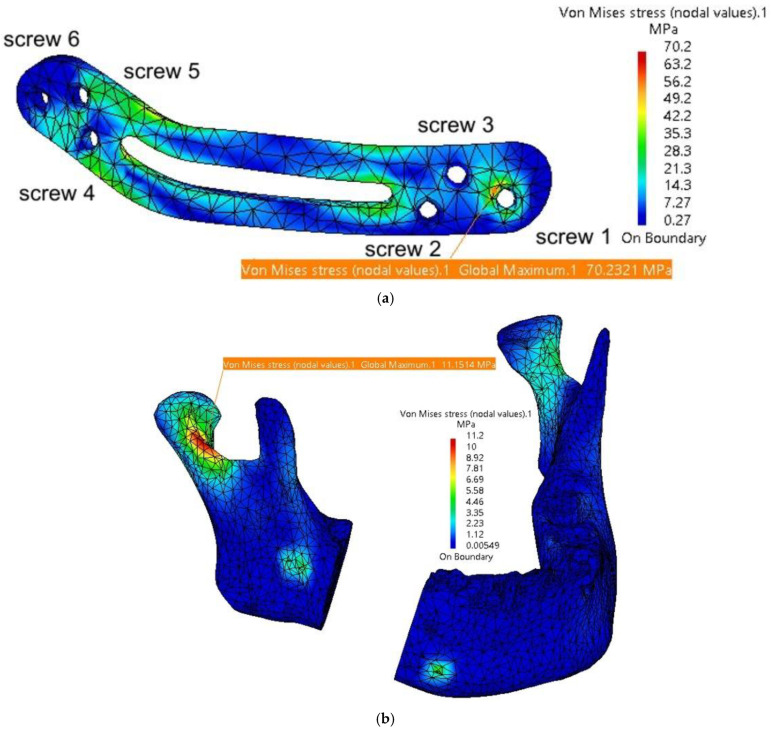
Stress distribution on the mandible in the assembly area: (**a**) stress distribution on the fixing plate in the assembly area with the six screws and the mandible; and (**b**) stress distribution on the mandible.

Figure 18 a illustrates the stress distribution within the plate model, with maximum stress concentrated in the regions around Screw 1 and Screw 4 at their contact points with the mandible. The mandible, depicted in Figure 18 b, shows maximum stress in the right condyle region, as well as around the screw assembly holes. Relatively high stresses are also observed in the two articular discs.

The FEM analysis reveals a relatively uniform distribution of stresses across the fixing plate and in the condylar regions of the mandible. Due to the rounded, streamlined design of the plate, no stress concentrators are observed at specific points. However, the central cutout, while potentially enhancing patient comfort, creates two areas of increased stress at its edges, thereby reducing the overall robustness of the plate. This cutout could be eliminated or replaced with several Ø2 mm holes to mitigate these stress concentrations.

The favorable performance of the plate is primarily attributed to its larger dimensions and the strategic placement of the screw holes. A reduction in the number of holes, although it might improve patient comfort, could result in incomplete fixation of the plate to the mandible, compromising its stability and functionality.

## 4. Discussion

This study evaluates three mandibular reconstruction strategies—titanium osteosynthesis plates, autogenous fibular grafts stabilized with titanium plates, and customized PEEK plates—using finite element analysis (FEA) to simulate biomechanical performance under masticatory loads. The objective is to present and recommend the optimal solutions for mandibular reconstruction following segmental resection due to ameloblastoma. 

To the best of the authors’ knowledge, this is one of the first studies to compare autogenous bone grafts with custom-designed titanium and PEEK plates [2]. By incorporating finite element analysis, this study provides a comprehensive evaluation of the biomechanical performance of these reconstruction strategies, emphasizing the importance of patient-specific customization. The findings highlight the potential of PEEK as a promising alternative to titanium and autogenous bone grafts, particularly in cases requiring reduced imaging artifacts and improved stress distribution.

The study comprises three main stages: the initial creation and optimization of the mandibular model, presented in detail in the video link provided as a guide/tutorial; the design of reconstruction plates with variable thicknesses; and the subsequent testing and evaluation of their biomechanical performance.

For the mandibular model, it could be observed that most components are discretized using parabolic finite elements. The type of finite elements used significantly influences the stresses and displacements observed in each component. Linear elements, characterized by their simpler geometry and nodal connections, result in linear displacement interpolation. When subjected to loads, linear elements exhibit linear deformation between the nodes. However, for complex geometries or surfaces, higher-order elements, such as parabolic or cubic elements, are recommended [29,30,31].

Parabolic elements, when loaded, exhibit deformation following parabolic equations due to the inclusion of additional nodes along the edges connecting the primary nodes. This enhances the accuracy of the simulation results at the cost of increased computational time [32], but the results are much closer to the real case.

To complement the results and conclusions presented in this study, a video demonstrating the stress and deformation distributions for the entire model across the three simulation scenarios is provided in the Supplementary Materials: https://youtu.be/qk0vEjPUeUs (accessed on 11 January 2025). This visual aid offers a comprehensive understanding of the biomechanical behavior of the evaluated reconstruction strategies.

The present study underscores the importance of material selection and plate design in achieving optimal clinical outcomes.

The titanium osteosynthesis plates demonstrated superior mechanical stability and resistance to deformation under masticatory loads, corroborating findings in similar studies. For instance, Kimura et al. [33] highlighted the efficacy of titanium plates in redistributing stress around screw fixations in reconstructed mandibles. However, the stress concentrations observed in our simulations near the screw fixation points are consistent with the potential for fatigue-related failures reported in other analyses of mandibular plates [34]. Despite these limitations, titanium plates remain a reliable choice for achieving primary stability in mandibular reconstruction.

The incorporation of an autogenous fibular graft stabilized with a titanium plate yielded superior outcomes in terms of stress distribution and structural support. The graft effectively absorbed stresses, reducing torsion and deformation in the titanium plate. This underscores the value of biologically integrated solutions in mandibular reconstruction. However, the need for an additional surgical procedure to harvest the graft remains a significant limitation, posing challenges for patient recovery and increasing the risk of donor site morbidity.

The incorporation of an autogenous fibular graft significantly enhanced the biomechanical outcomes, particularly in stress distribution and structural support. This aligns with Lang et al. [35], who demonstrated the biomechanical advantages of fibular free flaps due to their anatomical compatibility and high vascularization. However, the additional surgical intervention required for graft harvesting presents challenges, as noted by Kang et al. [36], particularly in patients with limited donor site availability or compromised healing capacity. The severity of the defect can serve as a clinical criterion for selecting the appropriate method of autogenous bone transplantation. For smaller segmental mandibular defects, such as the one presented in our clinical scenario, nonvascularized bone flap grafts are typically employed. In contrast, for larger defects, vascularized bone flap transplantation is the preferred approach [37,38].

PEEK plates emerged as a new and viable alternative, offering advantages such as radiolucency, reduced imaging artifacts, and mechanical properties closer to those of cortical bone. Their lower modulus of elasticity potentially reduce stress shielding effects compared to titanium plates. Similar observations were made by Mehle et al. [16] and Chen et al. [39], who emphasized the compatibility of PEEK for patient-specific designs in maxillofacial reconstruction. However, the simulations revealed stress concentrations around the central cutout and screw fixation points, necessitating careful design optimization to mitigate these effects. The localized stress concentrations observed in thinner PEEK plates underscore the need for further optimization of plate design to enhance their durability under physiological loads. This is consistent with findings by Lommen et al., who suggested that PEEK’s mechanical performance is heavily dependent on plate geometry and thickness [14]. While PEEK plates showed adequate performance at thicknesses of 1.8–2 mm, thinner plates risked plastic deformation under high loads, limiting their application in cases requiring extensive reconstruction.

Although our findings underscore the mechanical reliability and clinical potential of PEEK plates, recent advancements in material science suggest exciting opportunities for further enhancement through functionalization.

The PEEK material, modified with bioactive agents, has demonstrated promising results in improving implant integration and providing additional therapeutic benefits. For instance, surface modifications, such as the covalent attachment of bioactive peptides, have shown enhanced osteoblast adhesion and differentiation, promoting faster and more robust osseointegration [40]. Furthermore, the incorporation of bioactive ions, such as calcium and zinc, into PEEK matrices not only fosters osteogenesis but also modulates macrophage polarization to the M2 phenotype, a key factor in bone healing and regeneration [41].

Beyond surface chemistry, techniques such as plasma-immersion ion implantation and bioactive-coating deposition have also been employed to enhance the bioactivity of PEEK implants [42]. These functionalization strategies provide a dual advantage: improving the biological interface between the implant and bone while imparting properties such as antibacterial activity and enhanced biocompatibility.

In comparison, autogenous grafts with titanium plates provided the most favorable biomechanical performance, highlighting the importance of incorporating biological materials into reconstruction strategies (Table 5). However, in patients where grafting is contraindicated, customized PEEK plates offer a promising alternative due to their biocompatibility and tailored design capabilities [14,39].

Although our FEM model assumes static masticatory loads, previous studies have demonstrated that the mechanical performance of PEEK plates under dynamic cyclic loading remains robust, with stress levels well within the yield strength of the material, ensuring their suitability for clinical applications. 

Dynamic or fatigue simulations, while valuable, require significantly higher computational resources and may not yield additional insights in this context, as the stresses observed in our study were far below material failure thresholds; however, this will be explored in future research incorporating experimental models for validation.

Based on our findings, reconstructing the defect with autogenous bone is strongly recommended for such surgical interventions. However, this approach presents a significant challenge for the patient, as it requires an additional surgical procedure to harvest the graft, which, if chosen, would typically be performed concurrently with the segmental resection.

The present study has some limitations: Firstly, the simulations are based on idealized mandibular geometries, which may not fully replicate the anatomical variability observed in clinical cases. Additionally, the material properties of the mandible and plates were assumed to be homogenous and isotropic, which might not entirely capture the complex biomechanical behavior of biological tissues. Another limitation of this study includes the inherent challenges of FEA, such as cost, complexity, and the difficulty of accurately simulating dynamic interactions, particularly in edentulous patients with variable load distributions. This study focused on static simulations to provide clinically relevant findings, as dynamic analysis would introduce significant computational complexity without yielding additional meaningful insights in this context. 

Future research should focus on the long-term in vivo performance of these materials and their interaction with surrounding tissues. Additionally, advancements in additive manufacturing could further enhance the customization and mechanical properties of reconstruction plates, broadening their applicability in complex cases. The integration of patient-specific solutions through CAD/FEA technology represents a significant step forward in mandibular reconstruction, bridging the gap between functional restoration and patient-centered care.

## 5. Conclusions

The findings of this study underscore the biomechanical advantages and limitations of titanium, autogenous bone, and PEEK-based reconstruction strategies for segmental mandibular defects. Taking into account the study’s limitations, all three reconstruction options are viable, provided that the following minimum plate thicknesses are adhered to:

For titanium plates alone, a minimum thickness of 1.2–1.6 mm is recommended to ensure mechanical stability under masticatory loads.

When combined with an autogenous bone graft, the stress-absorbing properties of the graft allow titanium plates to perform adequately at a minimum thickness of 1.2 mm.

For PEEK plates, a minimum thickness of 1.8 mm is advised to avoid plastic deformation, with 2 mm offering greater safety under load conditions.

Titanium plates provide superior mechanical stability, making them the material of choice for cases requiring maximum load-bearing capacity. Autogenous bone grafts, on the other hand, enhance stress distribution and provide structural support, making them ideal for scenarios where improved biomechanical integration is essential. Customized PEEK plates emerge as a promising alternative due to their reduced stress shielding, biocompatibility, and radiolucency. However, their design requires optimization to address localized stress concentrations and ensure long-term durability.

The aim of this study is to provide surgeons with both theoretical and practical support to identify the optimal solution for each clinical case. By presenting clear guidelines and recommendations, the study seeks to assist clinicians in making informed decisions tailored to patient-specific needs. Furthermore, this research encourages the use of customized solutions to avoid the additional stress generated by intraoperative plate adjustments, particularly with titanium plates.

Future research should incorporate dynamic simulations and patient-specific modeling to refine these reconstruction approaches further and enhance their clinical applicability. These advancements will enable surgeons to achieve superior outcomes in mandibular reconstruction by leveraging the strengths of each material in conjunction with cutting-edge, customization technologies.

## Figures and Tables

**Table 1:** Key characteristics of the assembly components.

Component	Finite Element Size, mm	Absolute Sag, mm	Finite Element Type	Material
Mandible	1.5	0.8	Parabolic	Bone
Grafted autogenous bone	1.2	0.5	Parabolic	Bone
TMJ disc	0.8	0.1	Parabolic	Cartilage
Fixing plate titanium	1.5	0.2	Parabolic	Ti-Grade-4 (TI75A)
Fixing plate PEEK	1	0.5	Parabolic	PEEK
Screws	0.5	0.2	Linear	Ti-Grade-4 (TI75A)

TMJ = temporomandibular joint; absolute sag = the maximum distance between the original surface and the mesh approximation.

**Table 2:** Overview of the maximum stresses and displacements observed within the assembly mandible–titanium plate.

Component	Von Mises Stress, MPa	Translational Displacement Vector, mm	Component	Von Mises Stress, MPa	Translational Displacement Vector, mm
**Plate thickness = 2 mm**					
Mandible	19.3	1.9	Fixing plate	257	1.89
TMJ 1	2.58	0.197	Screw 2	10.7	-
TMJ 2	1.3	0.175	Screw 5	20.4	-
**Plate thickness = 1.8 mm**					
Mandible	19.4	1.89	Fixing plate	307	1.89
TMJ 1	2.63	0.199	Screw 2	11.8	-
TMJ 2	1.5	0.173	Screw 5	27.1	-
**Plate thickness = 1.6 mm**					
Mandible	19.3	1.88	Fixing plate	318	1.88
TMJ 1	2.67	0.201	Screw 2	15.5	-
TMJ 2	1.61	0.171	Screw 5	27.3	-
**Plate thickness = 1.4 mm**					
Mandible	19.2	1.87	Fixing plate	349	1.86
TMJ 1	2.69	0.204	Screw 2	18	-
TMJ 2	2.09	0.168	Screw 5	26.5	-
**Plate thickness = 1.2 mm**					
Mandible	19	1.87	Fixing plate	431	1.86
TMJ 1	2.69	0.204	Screw 2	24.2	-
TMJ 2	2.98	0.168	Screw 5	33.1	-

TMJ 1 = right temporomandibular joint (condyle–disc construct) on the resected site; TMJ 2 = left temporomandibular joint (condyle–disc construct); screws—numbered according to Figure 13.

**Table 3:** Overview of the maximum stresses and displacements observed within the assembly mandible–grafted bone–titanium plate.

Component	Von Mises Stress, MPa	Translational Displacement Vector, mm	Component	Von Mises Stress, MPa	Translational Displacement Vector, mm
**Plate thickness = 1.2 mm**					
Mandible	40.7	1.61	Fixing plate	12	1.53
Cartilage 1	2.62	0.118	Screw 2	2.11	1.41
TMJ 2	1.17	0.191	Screw 3	2.69	1.29
Bone insert	15.7	1.37	Screw 4	2.29	1.17
**Plate thickness = 1.4 mm**					
Mandible	40.3	1.61	Fixing plate	10.8	1.53
TMJ 1	2.68	0.118	Screw 2	2.09	1.41
TMJ 2	1.22	0.191	Screw 3	2.49	1.29
Bone insert	15.6	1.37	Screw 4	2.23	1.17
**Plate thickness = 1.6 mm**					
Mandible	40.2	1.61	Fixing plate	10.2	1.53
TMJ 1	2.64	0.118	Screw 2	1.77	1.41
TMJ 2	1.34	0.191	Screw 3	2.18	1.29
Bone insert	15.6	1.37	Screw 4	2.19	1.17
**Plate thickness = 1.8 mm**					
Mandible	40.1	1.61	Fixing plate	9.7	1.53
TMJ 1	2.78	0.118	Screw 2	1.79	1.41
TMJ 2	1.88	0.191	Screw 3	2.16	1.29
Bone insert	15.6	1.37	Screw 4	2.16	1.17

TMJ 1 = right temporomandibular joint (condyle–disc construct), on the resected site; TMJ 2 = left temporomandibular joint (condyle–disc construct); screws—numbered according to Figure 17a.

**Table 4:** Overview of the maximum stresses and displacements observed within the assembly mandible–PEEK plate.

Component	Von Mises Stress, MPa	Translational Displacement Vector, mm	Component	Von Mises Stress, MPa	Translational Displacement Vector, mm
**Plate thickness = 2 mm**					
Mandible	11.2	1.14	Screw 1 and 2	35 and 12.8	max 0.11
TMJ 1	2.98	0.2	Screw 3 and 4	24.2 and 26.3	max 0.09
TMJ 2	3.31	0.2	Screw 5	28.4	0.1
Fixing plate	70.2	1.12	Screw 6	18.2	0.09
**Plate thickness = 1.8 mm**					
Mandible	16.8	1.32	Screw 1 and 2	38 and 16.2	max 0.13
TMJ 1	3.1	0.2	Screw 3 and 4	28.4 and 32.1	max 0.11
TMJ 2	3.49	0.2	Screw 5	34.2	0.18
Fixing plate	81.29	1.29	Screw 6	12	0.1
**Plate thickness = 1.6 mm**					
Mandible	20.6	1.46	Screw 1 and 2	46 and 22.6	max 0.16
TMJ 1	3.48	0.22	Screw 3 and 4	34 and 42.4	max 0.14
TMJ 2	3.88	0.22	Screw 5	46	0.22
Fixing plate	92.7	1.38	Screw 6	16	0.12
**Plate thickness = 1.4 mm**					
Mandible	32.6	1.85	Screw 1 and 2	58 and 28.8	max 0.23
TMJ 1	3.79	0.24	Screw 3 and 4	41.9 and 49.5	max 0.2
TMJ 2	4.22	0.22	Screw 5	51.8	0.24
Fixing plate	130.4	1.7	Screw 6	28.4	0.17

TMJ 1 = right temporomandibular joint (condyle–disc construct), on the resected site; TMJ 2 = left temporomandibular joint (condyle–disc construct); screws—numbered according to Figure 18a.

**Table 5:** Comparison table of titanium plates and PEEK plates for mandibular reconstruction.

Characteristic	Titanium Plates	PEEK Plates
Mechanical strength	High mechanical stability, with superior load-bearing capacity.	Sufficient strength for physiological loads, but thinner plates (<1.8 mm) risk plastic deformation.
Elastic modulus	Very high (104 GPa), leading to stress shielding and reduced bone remodeling.	Closer to cortical bone (3.9 GPa), minimizing stress shielding and promoting better load distribution.
Imaging artifacts [43]	Causes significant streaking and blooming artifacts in CT and MRI imaging.	Radiolucent with minimal imaging artifacts, allowing for a clear postoperative assessment.
Weight	Denser and heavier, which may reduce patient comfort.	Lightweight, improving patient comfort and reducing load on surrounding tissues.
Plate thickness	Minimum thickness recommended: 1.2–1.6 mm for mechanical stability.	Minimum thickness recommended: 1.8–2.0 mm to avoid plastic deformation under physiological loads.
Biocompatibility	Highly biocompatible but can enhance radiation dose in adjacent tissues during radiotherapy.	Biocompatible and inert; does not interfere with radiotherapy or adjacent tissue assessment.
Manufacturing	3D printing or sintering; higher production cost.	Can be manufactured using additive techniques, offering reduced production costs and patient-specific design.

## Data Availability

The original contributions presented in this study are included in the article. Further inquiries can be directed to the corresponding author(s).

## Supplementary Materials

Video guides are available: https://youtu.be/DAt7YAio2A0 and https://youtu.be/qk0vEjPUeUs. Both videos are last checked and accessed on 11 January 2025

## References

[1] Sung H., Ferlay J., Siegel R.L., Laversanne M., Soerjomataram I., Jemal A., Bray F. (2021). Global Cancer Statistics 2020: GLOBOCAN Estimates of Incidence and Mortality Worldwide for 36 Cancers in 185 Countries. CA Cancer J. Clin..

[2] Aftabi H., Zaraska K., Eghbal A., McGregor S., Prisman E., Hodgson A., Fels S. (2024). Computational models and their applications in biomechanical analysis of mandibular reconstruction surgery. Comput. Biol. Med..

[3] Sozzi D., Cassoni A., De Ponti E., Moretti M., Pucci R., Spadoni D., Canzi G., Novelli G., Valentini V. (2022). Effectiveness of Resective Surgery in Complex Ameloblastoma of the Jaws: A Retrospective Multicenter Observational Study. Cancers.

[4] Gupta L., Mishra A., Gurav S.V., Dholam K., Pal A., Kumar A. (2024). Factors associated with mandibular deviation and proposed classification and treatment guidelines for applying mandibular guidance: A retrospective analysis of 185 patients with segmental mandibulectomy. J. Prosthet. Dent..

[5] Pyne J.M., Davis C.M., Kelm R., Bussolaro C., Dobrovolsky W., Seikaly H. (2023). Advanced mandibular reconstruction with fibular free flap and alloplastic TMJ prosthesis with digital planning. J. Otolaryngol.-Head Neck Surg..

[6] Weyh A.M., Fernandes R.P. (2021). Narrative review: Fibula free flap, indications, tips, and pitfalls. Front. Oral Maxillofac. Med..

[7] Hoffman G.R., Islam S., Eisenberg R.L. (2012). Microvascular reconstruction of the mouth, jaws, and face: Experience of an australian oral and maxillofacial surgery unit. J. Oral Maxillofac. Surg..

[8] Zeller A.N., Neuhaus M.T., Weissbach L.V.M., Rana M., Dhawan A., Eckstein F.M., Gellrich N.C., Zimmerer R.M. (2020). Patient-Specific Mandibular Reconstruction Plates Increase Accuracy and Long-Term Stability in Immediate Alloplastic Reconstruction of Segmental Mandibular Defects. J. Maxillofac. Oral Surg..

[9] Saunders J.R., Hirata R.M., Jaques D.A. (1990). Definitive mandibular replacement using reconstruction plates. Am. J. Surg..

[10] Kumar B.P., Venkatesh V., Kumar K.A.J., Yadav B.Y., Mohan S.R. (2015). Mandibular Reconstruction: Overview. J. Maxillofac. Oral Surg..

[11] Kitami R., Izumi M., Taniguchi M., Kozai Y., Sakurai T. (2024). Phantom study for CT artifacts of dental titanium implants and zirconia upper structures: The effects of occlusal plane angle setting and SEMAR algorithm. Oral Radiol..

[12] Huber F.A., Sprengel K., Müller L., Graf L.C., Osterhoff G., Guggenberger R. (2021). Comparison of different CT metal artifact reduction strategies for standard titanium and carbon-fiber reinforced polymer implants in sheep cadavers. BMC Med. Imaging.

[13] Midthun P., Kirkhus E., Østerås B.H., Høiness P.R., England A., Johansen S. (2023). Metal artifact reduction on musculoskeletal CT: A phantom and clinical study. Eur. Radiol. Exp..

[14] Lommen J., Schorn L., Sproll C., Kübler N.R., Nicolini L.F., Merfort R., Dilimulati A., Hildebrand F., Rana M., Greven J. (2022). Mechanical Fatigue Performance of Patient-Specific Polymer Plates in Oncologic Mandible Reconstruction. J. Clin. Med..

[15] Panayotov I.V., Orti V., Cuisinier F., Yachouh J. (2016). Polyetheretherketone (PEEK) for medical applications. J. Mater. Sci. Mater. Med..

[16] Mehle K., Eckert A.W., Gentzsch D., Schwan S., Ludtka C.M., Knoll W. (2016). Evaluation of a New PEEK Mandibular Reconstruction Plate Design for Continuity Defect Therapy by Finite Element Analysis. Int. J. New Technol. Res..

[17] Vayvada H., Mola F., Menderes A., Yilmaz M. (2006). Surgical Management of Ameloblastoma in the Mandible: Segmental Mandibulectomy and Immediate Reconstruction With Free Fibula or Deep Circumflex Iliac Artery Flap (Evaluation of the Long-Term Esthetic and Functional Results). J. Oral Maxillofac. Surg..

[18] Gasparro R., Giordano F., Campana M.D., Aliberti A., Landolfo E., Dolce P., Sammartino G., di Lauro A.E. (2024). The Effect of Conservative vs. Radical Treatment of Ameloblastoma on Recurrence Rate and Quality of Life: An Umbrella Review. J. Clin. Med..

[19] Chen C., Batstone M., Taheri T., Johnson N. (2024). Surgical Resection and Reconstruction of Ameloblastoma: A 13-Year Retrospective Review. J. Oral Maxillofac. Surg..

[20] Xue R., Lai Q., Xing H., Zhong C., Zhao Y., Zhu K., Deng Y., Liu C. (2024). Finite element analysis and clinical application of 3D-printed Ti alloy implant for the reconstruction of mandibular defects. BMC Oral Health.

[21] Shen Y.W., Tsai Y.S., Hsu J.T., Shie M.Y., Huang H.L., Fuh L.J. (2022). Biomechanical Analyses of Porous Designs of 3D-Printed Titanium Implant for Mandibular Segmental Osteotomy Defects. Materials.

[22] Kucukguven M.B., Akkocaolu M. (2020). Finite element analysis of stress distribution on reconstructed mandibular models for autogenous bone grafts. Technol. Health Care.

[23] Melville J.C., Mañón V.A., Arribas A.R., Wong M.E. (2021). Custom 3D printed titanium reconstruction plate with in-situ tissue engineering for the reconstruction and dental rehabilitation of a severely infected atrophic mandible. A review of technique. Dent. Rev..

[24] Chakraborty S., Guha R.P., Naskar S., Banerjee R. (2022). Custom-Made 3D Titanium Plate for Mandibular Reconstruction in Surgery of Ameloblastoma: A Novel Case Report. Surg. Tech. Dev..

[25] Tanaka E. (2021). Biomechanical and tribological properties of the temporomandibular joint. Front. Oral Maxillofac. Med..

[26] De Stefano M., Ruggiero A. (2024). A Critical Review of Human Jaw Biomechanical Modeling. Appl. Sci..

[27] Ghionea I.G., Vatamanu O.E.B., Cristescu A.M., David M., Stancu I.C., Butnarasu C., Cristache C.M. (2023). A Finite Element Analysis of a Tooth-Supported 3D-Printed Surgical Guide without Metallic Sleeves for Dental Implant Insertion. Appl. Sci..

[28] Pinheiro M., Willaert R., Khan A., Krairi A., Van Paepegem W. (2021). Biomechanical evaluation of the human mandible after temporomandibular joint replacement under different biting conditions. Sci. Rep..

[29] Ghionea I.G. (2024). CATIA V5 Practical Studies Using Finite Element Analysis.

[30] Ashcroft I.A., Mubashar A. (2011). Numerical Approach: Finite Element Analysis. Handbook of Adhesion Technology.

[31] Tang Y., Qing H. (2024). Finite element formulation for higher-order shear deformation beams using two-phase local/nonlocal integral model. Arch. Appl. Mech..

[32] Hilber N., Reichmann O., Schwab C., Winter C. (2013). Finite Element Methods for Parabolic Problems. Computational Methods for Quantitative Finance: Finite Element Methods for Derivative Pricing.

[33] Kimura A., Nagasao T., Kaneko T., Tamaki T., Miyamoto J., Nakajima T. (2006). Adaquate fixation of plates for stability during mandibular reconstruction. J. Cranio-Maxillofac. Surg..

[34] Seebach M., Theurer F., Foehr P., von Deimling C., Burgkart R., Zaeh M.F. (2018). Design of Bone Plates for Mandibular Reconstruction Using Topology and Shape Optimization. Advances in Structural and Multidisciplinary Optimization.

[35] Lang J.J., Bastian M., Foehr P., Seebach M., Weitz J., Von Deimling C., Schwaiger B.J., Micheler C.M., Wilhelm N.J., Grosse C.U. (2021). Improving mandibular reconstruction by using topology optimization, patient specific design and additive manufacturing?—A biomechanical comparison against miniplates on human specimen. PLoS ONE.

[36] Kang J., Zhang J., Zheng J., Wang L., Li D., Liu S. (2021). 3D-printed PEEK implant for mandibular defects repair—A new method. J. Mech. Behav. Biomed. Mater..

[37] Batstone M.D. (2018). Reconstruction of major defects of the jaws. Aust. Dent. J..

[38] Likhterov I., Roche A.M., Urken M.L. (2019). Contemporary Osseous Reconstruction of the Mandible and the Maxilla. Oral Maxillofac. Surg. Clin. North Am..

[39] Chen L., Gao L., Cui H., Guo X., Han J., Liu J., Yao Y. (2024). Finite element comparison of titanium and polyetheretherketone materials for mandibular defect reconstruction. Am. J. Transl. Res..

[40] Cassari L., Balducci C., Messina G.M.L., Iucci G., Battocchio C., Bertelà F., Lucchetta G., Coward T., Silvio L.D., Marletta G. (2024). Polyetheretherketone Double Functionalization with Bioactive Peptides Improves Human Osteoblast Response. Biomimetics.

[41] Jones E.A., Sigurjónsson Ó.E., Weng X., Zhu W., Chai H., Wang W., Yuan X., Zhu C. (2022). Bio-Activated PEEK: Promising Platforms for Improving Osteogenesis through Modulating Macrophage Polarization. Bioengineering.

[42] Dondani J.R., Iyer J., Tran S.D. (2023). Surface Treatments of PEEK for Osseointegration to Bone. Biomolecules.

[43] Lommen J., Schorn L., Sproll C., Haussmann J., Kübler N.R., Budach W., Rana M., Tamaskovics B. (2022). Reduction of CT Artifacts Using Polyetheretherketone (PEEK), Polyetherketoneketone (PEKK), Polyphenylsulfone (PPSU), and Polyethylene (PE) Reconstruction Plates in Oral Oncology. J. Oral Maxillofac. Surg..

